# Practical Considerations and Recommendations for “a Revised Hippocratic Oath for the Era of Digital Health”

**DOI:** 10.2196/43383

**Published:** 2022-10-26

**Authors:** Rachele Hendricks-Sturrup, Maryam Nafie, Christine Lu

**Affiliations:** 1 Duke-Margolis Center for Health Policy Washington, DC United States; 2 Harvard Pilgrim Health Care Institute Boston, MA United States; 3 Department of Population Medicine Harvard Medical School Boston, MA United States

**Keywords:** digital health, Hippocratic Oath, eHealth, ethics, digital divide

## Abstract

The Hippocratic Oath (the “Oath”) is a longstanding body of ethical tenets that have undergone several amendments to accommodate changes and evolutions in the practice of medicine. In their recent perspective entitled, “A Revised Hippocratic Oath for the Era of Digital Health,” Meskó and Spiegel offered proposed amendments to the Oath to address both challenges and needs that follow digital health implementation in clinical practice. In this commentary, we offer additional thoughts and considerations to Meskó and Spiegel’s proposed amendments to accomplish two goals: (1) reflect on the shared goals and values of all digital health stakeholders and (2) drive home the focus on affirming patient choice, autonomy, and respect.

## Introduction

History repeatedly reveals that as society faces accelerated change brought by industrial revolution, both institutions and those operating within them must adapt to and endure such changes to survive. Health care institutions, and the practice of medicine in general, are no exception, as we see today in the digital health era.

Meskó and colleagues [1,2] have defined the “digital health era” as today’s era in which a “cultural transformation of how disruptive technologies that provide digital and objective data accessible to both health care providers and patients leads to an equal-level doctor-patient relationship with shared decision-making and the democratization of care.” Therefore, this definition naturally sparks a multilayered discussion around the ethics of digital health implementation in clinical practice. The longstanding Hippocratic Oath (the “Oath”), for example, and as discussed recently by Meskó and Spiegel [3] in their latest perspective entitled, “A Revised Hippocratic Oath for the Era of Digital Health,” is one such level at which the basic ethical tenets of health care can or should be reimagined.

In this commentary, we aim to accomplish two goals in response to Meskó and Spiegel’s [3] proposed changes to the Oath: (1) reflect on the shared goals and values of all digital health stakeholders and (2) drive home the focus on affirming patient choice, autonomy, and respect.

## Key Considerations and Recommendations

As the medical community contemplates Meskó and Spiegel’s [3] proposed new text (in brackets) for the digital health era, we offer line-by-line comments and considerations that serve to encourage deeper thought around the real-world implications for a potentially revised Oath.

I will respect the hard-won scientific gains of those physicians, [researchers, and patients] in whose steps I walk, and gladly share such knowledge as is mine with those who are to follow.

Today, health care is accelerated by the rapid development and implementation of electronic health records, patient-provider portals, mobile health apps, wearable biosensors, artificial intelligence, social media platforms, etc, in brick-and-mortar, remote, and virtual reality settings. Those responsible for such rapid developments, including their clinical implementation, are clinicians in general (not just physicians), inventors, patients, insurers, technology developers, venture capitalists, and many other stakeholders. Their collective hard-won scientific gains should be acknowledged in the Oath to not just give credit as due but also offer transparency around who is involved in the scientific advancements that drive health care in the 21st century.

I will apply, for the benefit of [the healthy and] the sick, all measures [that] are required, avoiding those twin traps of overtreatment and therapeutic nihilism.

Healthy patients and patients with low health care utilization, for whatever reason, often lack a digital footprint in health care settings (ie, lack an electronic medical record history). Therefore, all patients with seemingly low or lack of health care utilization may erroneously be interpreted by artificial intelligence or machine learning algorithms that process digital health data (eg, electronic medical record data) as “healthy.” This is especially true for noncentralized health care systems like those within the United States, where patients may either lack a digital health record altogether due to a lack of insurance status or have fragmented digital health records due to multiple changes in employer-sponsored insurance coverage. This may lead to negative consequences, including inappropriate recommendations for patients based on incorrect estimates of health care utilization patterns. Therefore, it is important to consider potential algorithmic errors that accompany sole or vast reliance on digital health tools in lieu of adopting a more holistic and interpersonal approach to patient care. Additionally, it is important to contemplate the role that digital health plays in triggering illness in seemingly healthy individuals (eg, social media contributing to the onset of depression, anxiety due to overscreening, etc).

I will remember that there is an art to medicine as well as science, and that warmth, sympathy, and understanding may outweigh the surgeon’s knife, the chemist’s drug [or the programmer’s algorithm].

Patients will increasingly gain digital identities across a growing range of sensitive health care scenarios that may reach beyond any programmer’s algorithm (eg, cancer radiology, mental health or substance abuse, family planning, etc). Therefore, we argue that empathy is the utmost imperative for the Oath to ensure that patients are not treated merely as data subjects.

[I will treat my patients in an equal-level partnership, and] I will not be ashamed to say “I know not,” nor will I fail to call in my colleagues when the skills of another are needed for a patient’s recovery.

Patients are increasingly cost-conscientious and, therefore, have growing needs and demands for cost-related conversations with clinicians. Moreover, equal-level partnerships in patient-provider settings are complicated by histories of systemic racism that have created power imbalances between clinicians and patients. The paternalistic nature of digital health surveillance complicates this matter, making equal-level partnerships potentially illusory to those who have been subjected to negative experiences in the pursuit of health care. Last but not least, an enormous amount of information and power asymmetry exists today between patient communities and health systems, which contributes to health disparities and poor clinical or health outcomes for certain groups of people. Therefore, embedding concepts of equal-level partnership in the Oath may render it infeasible in practice due to long-standing biases and inequities that are deeply rooted in many health care systems everywhere.

I will respect the privacy of my patients [and their data], for their problems are not disclosed to me that the world may know.

A vast amount of digital health data, particularly in the consumer health space and marketplace, fall outside of the scope of existing laws that may protect patient privacy (eg, the US Health Insurance Portability and Accountability Act). In addition, health information privacy is often a matter of context, whereas digital data that are presumably non–health-related can become health-related depending on when, where, why, and by whom the data are collected and used (eg, ridesharing and geolocation apps may collect data about patients’ whereabouts around or outside of a medical campus). Furthermore, patients may unknowingly generate data that can become leveraged in the data marketplace or another venue without the patients’ consent. Therefore, clinicians should fully consider the privacy practices of digital software or device vendors, health systems, and others to determine whether these proposed changes to the Oath are truly feasible in practice. This is especially given that patients generate large amounts of data as health consumers in general, causing clinicians to rarely encounter or use such vast quantities of data in medical practice.

I will remember that I do not treat a fever chart, a cancerous growth, [a data point, or an algorithm’s suggestion,] but a human being.

Patients may knowingly or unknowingly become data subjects. While data are usually averaging a population, clinicians should always focus on the individual patient sitting in front of them. Therefore, today it is critical to create and pave a clear path toward reimagining and reaffirming patient autonomy and respect across all clinical practice areas and settings in which digital health is or may become implemented.

## Acknowledging Shared Goals and Patient Choice, Autonomy, and Respect in the Digital Era

Although the Oath was developed in ancient Greece, Meskó and Spiegel [3] noted that the Oath has undergone several amendments, with perhaps the most recent being led by the World Medical Association in 1948, resulting in the Declaration of Geneva [4]. Importantly, the Declaration of Geneva helped drive greater acknowledgment toward shared goals and values among clinical stakeholders, as well as patient autonomy and dignity. These goals are congruent with and complement our goals for this commentary and should, therefore, not be remiss.

## References

[1] Mesko B, Győrffy Z (2019). The rise of the empowered physician in the digital health era: viewpoint. J Med Internet Res.

[2] Meskó Bertalan, Drobni Z, Bényei Éva, Gergely B, Győrffy Z (2017). Digital health is a cultural transformation of traditional healthcare. Mhealth.

[3] Meskó Bertalan, Spiegel B (2022). A revised Hippocratic Oath for the era of digital health. J Med Internet Res.

[4] WMA Declaration of Geneva. World Medical Association.

